# Learning to silence saccadic suppression

**DOI:** 10.1073/pnas.2012937118

**Published:** 2021-02-01

**Authors:** Chris Scholes, Paul V. McGraw, Neil W. Roach

**Affiliations:** ^a^Visual Neuroscience Group, School of Psychology, University of Nottingham, Nottingham NG7 2RD, United Kingdom

**Keywords:** saccadic suppression, perceptual learning, microsaccades

## Abstract

Perceptual stability is facilitated by a decrease in visual sensitivity during rapid eye movements, called saccadic suppression. While a large body of evidence demonstrates that saccadic programming is plastic, little is known about whether the perceptual consequences of saccades can be modified. Here, we demonstrate that saccadic suppression is attenuated during learning on a standard visual detection-in-noise task, to the point that it is effectively silenced. Across a period of 7 days, 44 participants were trained to detect brief, low-contrast stimuli embedded within dynamic noise, while eye position was tracked. Although instructed to fixate, participants regularly made small fixational saccades. Data were accumulated over a large number of trials, allowing us to assess changes in performance as a function of the temporal proximity of stimuli and saccades. This analysis revealed that improvements in sensitivity over the training period were accompanied by a systematic change in the impact of saccades on performance—robust saccadic suppression on day 1 declined gradually over subsequent days until its magnitude became indistinguishable from zero. This silencing of suppression was not explained by learning-related changes in saccade characteristics and generalized to an untrained retinal location and stimulus orientation. Suppression was restored when learned stimulus timing was perturbed, consistent with the operation of a mechanism that temporarily reduces or eliminates saccadic suppression, but only when it is behaviorally advantageous to do so. Our results indicate that learning can circumvent saccadic suppression to improve performance, without compromising its functional benefits in other viewing contexts.

## Significance

Sensory systems often suppress self-generated sensations in order to discriminate them from those arising in the environment. The suppression of visual sensitivity during rapid eye movements is well established, and although functionally beneficial most of the time, it can limit the performance of certain tasks. Here, we show that with repeated practice, mechanisms that suppress visual signals during eye movements can be modified. People trained to detect brief visual patterns learn to turn off suppression around the expected time of the target. These findings demonstrate an elegant form of plasticity, capable of improving the visibility of behaviorally relevant stimuli without compromising the wider functional benefits of suppression.

Humans continuously sample the external world using frequent and rapid gaze shifts called saccades, which cause the image of the visual scene to sweep across the retina. The fact that we remain unaware of these frequent disruptions to visual input and maintain a stable perception of the world has intrigued generations of vision scientists (1–9). While early researchers attributed the lack of awareness of intrasaccadic stimulation to a form of central anesthesia (6), it is most commonly associated with the phenomenon of saccadic suppression—a reduction in the visibility of brief flashes presented around the time of a saccade. A large number of studies spanning more than a century have reported changes in the threshold for, or probability of detecting, brief perisaccadic stimuli. These effects have been demonstrated for different classes of saccadic eye movements and under a range of experimental conditions; for example, with large reactive saccades initiated under instruction, smaller spontaneous saccades during attempted fixation (10–12), targets presented on clear (11, 13) and textured (10, 14, 15) backgrounds, and in both the central (16, 17) and peripheral (11, 14, 18, 19) visual field. Despite widespread agreement that saccadic suppression is a robust phenomenon, consensus regarding its underlying mechanisms has proved elusive. During natural viewing, it is likely that the postsaccadic visual scene acts to mask the intrasaccadic image, which has been blurred by its rapid translation across the retina (4, 20–24). However, some researchers have argued for an active form of suppression, triggered by an extraretinal signal or corollary discharge of the saccadic eye movement (refs. 8 and 25–28; for counter arguments, see refs. 3, 4, and 23).

Saccades were classically considered to be highly stereotyped movements, reflecting the fact that they have relatively stable kinematic properties that are resistant to voluntary control and modification by training (29, 30). However, it is becoming increasingly clear that most characteristics of saccades can be modified. Evidence for saccadic plasticity comes from a variety of sources. Rather than being fixed, the “main sequence” relationship between saccade velocity and amplitude can be manipulated by visual stimulation (31) or reward (32, 33). When saccadic targets are consistently displaced by an experimenter during flight, individuals quickly adapt the amplitude (34–36) or direction (37) of future saccades to minimize landing errors. Repeated training on oculomotor tasks has been shown to decrease saccadic reaction times and increase the frequency of a variety of saccade types (38–41). In addition, close examination of eye movements has revealed that saccade production adjusts to meet current behavioral goals. During the threading of a needle, for example, small spontaneous saccades move the eye regularly between the tip of the thread and the eye of the needle in order to estimate relative alignment between the two (42). In addition, saccade rate and amplitude distributions are influenced by a variety of factors such as task complexity (43, 44), whether an individual is performing free-viewing or visual search (45), the size of the visual scene (46), and how informative an image region is (47).

In contrast to the growing literature documenting different forms of oculomotor plasticity, far less is known about the extent to which the perceptual consequences of saccades can be modified. This is partly due to ongoing debate over the relative contribution of active (extraretinal) and passive (masking) mechanisms to saccadic suppression, which has dominated much of the work in this area. Given its hypothesized functional role in maintaining visual stability across eye movements, it is tempting to assume that saccadic suppression must be a stable, perhaps even hard-wired effect that is impervious to training. Empirical validation of this assumption is lacking, however, as most studies aggregate perisaccadic visual judgements (often in a small number of well-trained observers) across multiple testing sessions. Two recent studies have reported learned improvements in visual task performance following training with stimuli consistently presented before (48) or during (49) saccades. While these findings demonstrate that perceptual learning is possible around the time of saccades, the role of suppression in this process remains unclear. Is saccadic suppression impervious to learning, placing an upper limit on the amount of improvement that is achievable with training? Or can learning modify saccadic suppression in a manner that actively contributes to improvements in sensory performance?

To address these questions, we measured visual sensitivity to a brief peripheral target stimulus embedded in luminance noise. This task consistently shows large improvements in performance with practice, and variants have been used extensively to investigate the characteristics and mechanisms of perceptual learning. Rather than direct subjects to make large saccades around the time of stimulus presentation, we instead exploited spontaneously occurring saccades during attempted fixation. This approach had several advantages. From a practical perspective, it enabled us to make use of a standard perceptual learning paradigm, with the only addition being noninvasive monitoring of eye position. Moreover, it avoided the conflict of having to instruct subjects to perform the task as accurately as possible, while also making eye movements that would likely impair their ability to do so. While the majority of suppression studies focus on a small number of highly trained individuals, we were able to instead use a larger group of completely naive subjects without the need for any eye movement training. Although saccadic suppression has generally been investigated using large voluntary saccades, a body of evidence suggests that the visual consequences of fixational and voluntary saccades are comparable (12, 25, 26, 50–53), consistent with a view that fixational saccades are part of a saccadic continuum that is simply delineated by the magnitude of the eye movement (28, 51, 54–57).

## Results

### Perceptual Learning Is Accompanied by a Reduction of Saccadic Suppression.

**Fig. 1.**
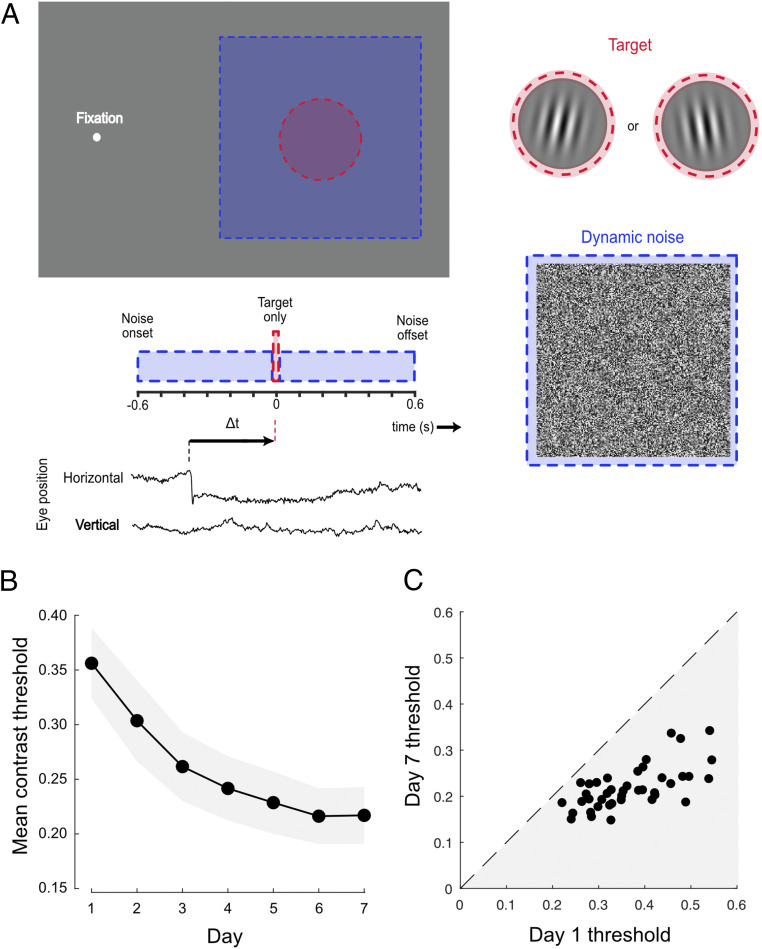
Robust learning on a conventional visual detection-in-noise task. (*A*) Subjects fixated on a dot and were required to indicate the orientation (±10° of vertical) of a brief peripheral grating embedded in dynamic white noise. The timing of the target and noise was identical on each trial. Individuals spontaneously made small fixational saccades (see eye position trace), allowing sorting of trials depending on the time of the target relative to the nearest saccade (Δ*t*). (*B*) Mean contrast thresholds across subjects decreased as a function of training day. Each subject completed five contrast staircases of 100 trials on each day. Error bars are 95% CIs across subjects. (*C*) All subjects (represented as dots) demonstrated learning, having lower contrast thresholds on day 7 than on day 1.

We employed a conventional task to investigate visual perceptual learning, requiring discrimination of the orientation of peripherally presented target gratings embedded in luminance noise (Fig. 1*A*). Subjects (*n* = 44) were instructed to maintain fixation, but spontaneously made frequent, small-amplitude saccades that were detected with a high-speed video eye tracker (*Materials and Methods*). Across 7 days of testing, trial-by-trial feedback was provided and target contrast was manipulated via a series of adaptive staircases. To confirm that training was effective in improving performance, we first calculated contrast thresholds using all of the trials completed by each subject on each day. In line with previous studies (58, 59), we found clear evidence of learning, with mean contrast thresholds falling exponentially across the training period (Fig. 1*B*). These improvements were robust in all subjects (Fig. 1*C*) and exhibited characteristic learning dynamics both within and across daily training sessions (60, 61) (*SI Appendix*, Fig. S1*A*).

**Fig. 2.**
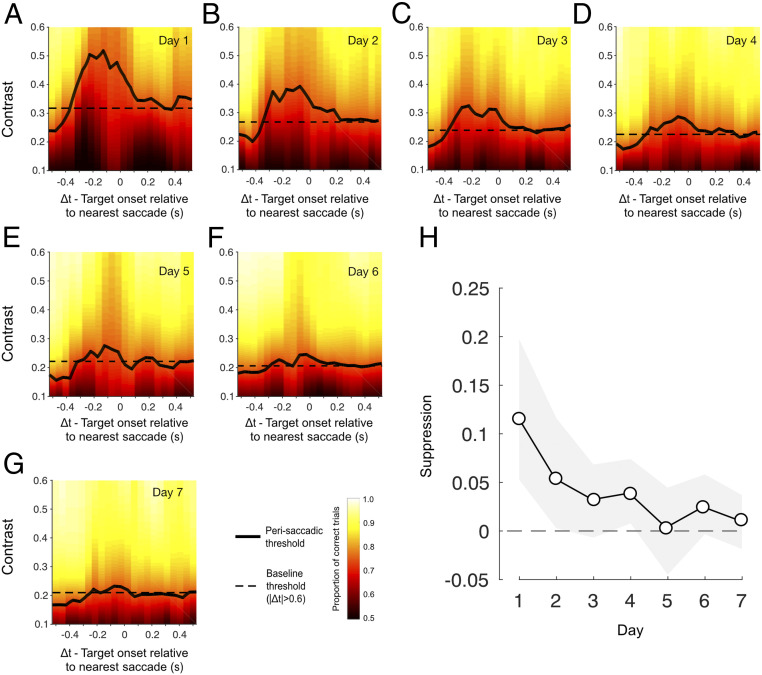
Saccadic suppression reduces over the course of training. (*A*–*G*) Perisaccadic thresholds (solid black lines) were raised in a broad time window around the saccade on day 1 (relative to no-saccade baseline trials) but systematically decreased as the week progressed. Thresholds were derived by binning trials according to the timing of the target onset relative to the nearest saccade (sliding 150-ms window) and fitting the resulting psychometric functions. Fitted proportion correct is indicated by the background color, with “warmer” colors signifying better performance. Baseline performance, derived from all trials without a saccade within 600 ms of the target stimulus, is indicated by the dashed horizontal lines. (*H*) Suppression magnitude, computed as the threshold from a broad suppression window (−100 ms < Δ*t* < 100 ms) minus the baseline threshold (|Δ*t*| < 0.6 s), as a function of training day. Error bars indicate bootstrapped 95% CIs.

To quantify changes in visual sensitivity around the time of saccadic eye movements, trial data were pooled across subjects and binned according to the timing of the target stimulus relative to the nearest saccade (Δ*t*, Fig. 1*A*). Fig. 2 *A*–*G* shows perisaccadic contrast thresholds (solid lines) plotted alongside a daily baseline (dashed line) derived from trials in which no saccade occurred within 600 ms of the target stimulus. Strong saccadic suppression was evident on day 1 (Fig. 2*A*), with marked elevation of thresholds on trials where target presentation occurred within a broad time window around a saccade. However, as the week of training progressed, saccadic suppression systematically reduced to the point that perisaccadic performance became indistinguishable from baseline (Fig. 2 *B*–*G*). To summarize these changes, a single estimate of suppression magnitude was calculated for each day by subtracting baseline thresholds from those obtained using a broad perisaccadic time window (100 ms before saccade onset to 100 ms after). As shown in Fig. 2*H*, suppression elevated contrast thresholds by ∼0.12 on day 1, but its effect rapidly approached zero as the week progressed. Put simply, perceptual learning led to a near-complete silencing of saccadic suppression.

As is typical in perceptual learning experiments, there were differences in absolute performance level across individual subjects. Subjects also differed in their saccade rate (0.2 to 2 saccades per second across subjects; mean, 0.8), which had the consequence that individuals could make unequal contributions to each of our group performance metrics. To ensure that our findings were not distorted by this combination of factors, we performed a series of permutation tests to estimate the suppression that would be expected given the number of trials that each subject contributed to each perisaccadic and baseline time window. Our results indicated that observed suppression on day 1 was larger than the most conservative (alpha = 0.001) subject-matched estimate, and decreased in magnitude and significance as the week progressed (*SI Appendix*, Fig. S2). We also conducted a subject-matched permutation test to assess the changes in suppression magnitude with training, shown in Fig. 2*H* (*Materials and Methods*). This confirmed a significant 90.3% attenuation in suppression from day 1 to day 7 (*P*_2-tailed_ < 0.001).

### Silencing of Suppression Is Not Due to Learning-Related Changes in Saccade Parameters.

**Fig. 3.**
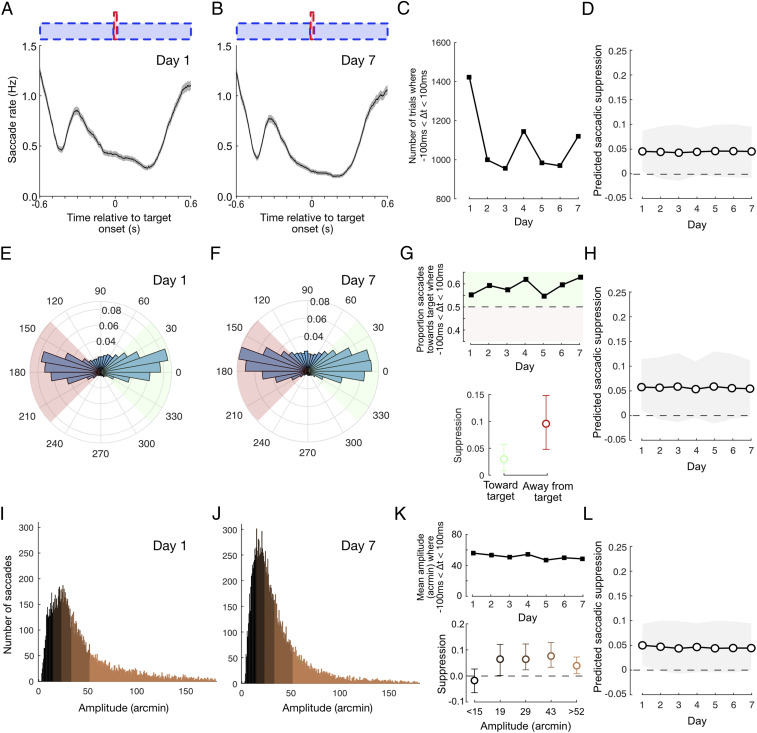
Suppression reduction is not due to learning-related changes in saccade parameters. (*A* and *B*) Saccade rate fluctuates as a function of noise and target onsets, both of which are highly predictable. This behavior arises quickly within the first day of training and continues to develop as training progresses. (*C*) The number of trials with saccade timings falling within the suppression interval (−100 ms < Δ*t* < 100 ms) drops after day 1. (*D*) Accounting for the change in trial numbers, suppression is predicted to be flat across the week, indicating that the reduction in suppression is not due to rate changes (see *Materials and Methods* for further details). (*E* and *F*) The majority of saccades were oriented horizontally either toward or away from the target. (*G*) The proportion of target-oriented saccades during the suppression interval increased modestly during training (*Top*) and suppression was slightly lower for target-oriented saccades (*Bottom*). (*H*) Suppression predicted from the change in orientation ratio decreased only slightly across the week and did not account for the magnitude of suppression reduction that we observed. (*I* and *J*) Histograms of saccade amplitude on days 1 and 7. (*K*) Median saccade amplitude during the suppression interval dropped slightly across the week (*Top*), and there were differences in suppression magnitude depending on saccade amplitude (*Bottom*). (*L*) Suppression predicted from the change in saccade amplitude was flat across the week, indicating that the reduction in suppression is not due to changes in amplitude. All error bars show 95% CIs.

During the course of the training week, the rate, orientation, and amplitude of saccades displayed subtle changes. We next assessed whether these changes may have indirectly affected our measures of saccadic suppression. The rate at which subjects made fixational saccades was not static but fluctuated relative to noise and target onsets (Fig. 3*A*). This is unsurprising given substantial evidence for the stimulus-related rate signature (62–65) and the reduction in saccade rate associated with predictable stimulus timing (66). This rate modulation developed within the first day of training but changed as the week continued, such that the number of saccades being made around target appearance decreased (Fig. 3 *B* and *C*). To assess whether this rate change might contribute to the reduction in saccadic suppression, we estimated the changes that would be expected solely based on the number of saccades falling within suppression and baseline windows on each day (*Materials and Methods*). This produced a predicted suppression profile (Fig. 3*D*) that was flat across the week, indicating that the reduction in suppression (Fig. 2*H*) was not a by-product of changes in the number or timing of saccades.

We were also careful to ensure that our findings were not specific to situations where the appearance of the target could have triggered a saccade. Restricting our analysis to an exclusively postsaccadic interval (0 to 100 ms) produced a comparable pattern to that shown in Fig. 2*H* (*SI Appendix*, Fig. S3), confirming that silencing of saccadic suppression could not be a by-product of changes in the stimulus-evoked rate signature. It should be noted, however, that performance-linked changes in saccade rate may have contributed to the broad presaccadic extent of suppression and tendency for thresholds to appear lower than baseline when targets were presented ∼400 to 600 ms prior to saccade onset (Fig. 2 *A*–*G* and see *SI Appendix*, *Supplementary Text 1*, for further discussion).

Fixational saccades were generally oriented horizontally, with the orientation distribution remaining relatively constant across the week (distributions from day 1 and 7 are displayed in Fig. 3 *E* and *F*, respectively). During the suppression window, subjects were marginally more likely to make a saccade oriented toward the target on day 1 and the proportion of target-oriented saccades increased slowly but systematically as the week progressed, so that nearly 65% of saccades were toward the target on day 7 (Fig. 3 *G*, *Top*). Given that suppression was greater when all saccades directed away from the target were considered (than those toward, Fig. 3 *G*, *Bottom*), we questioned whether the rebalancing of saccade orientation could explain our reported reduction in saccadic suppression. Suppression predicted from permutations in which the daily ratio of each orientation group was matched to the data did exhibit a decrease across the week, but this decrease was so slight that it is barely visible (Fig. 3*H*) when plotted on the same axes as the original suppression profile (Fig. 2*H*). We conclude that the reduction in suppression across the week is not explained by changes in the balance of saccade orientation.

Saccade amplitudes decreased slightly over the course of the week (Fig. 3 *I*–*K*, *Top*), with the mean amplitude dropping from just over 40 arcmin (2/3 degree of visual angle) on day 1 to just below 40 arcmin on day 7. Suppression was greatest for medium-sized saccades (Fig. 3 *K*, *Bottom*) but decreased for both small saccades (<15 arcmin) and large saccades (>52 arcmin). However, when the daily contribution of saccades with different amplitudes was matched, permutation tests predicted that suppression would be constant across the week (Fig. 3*L*). Therefore, the reduction in suppression was not due to changes in the amplitude of saccades across the week.

As a final check, we conducted a permutation test of the reduction in observed suppression from day 1 to day 7 that explicitly controls for the combined effects of changes in the number, direction, and amplitude of saccades (*SI Appendix*, *Supplementary Text 2*). This analysis provided confirmation that the attenuation in suppression remains statistically significant (*P*_2-tailed_ = 0.018), even when changes in joint saccade statistics are accounted for.

### Silencing of Suppression Generalizes to Untrained Stimuli.

**Fig. 4.**
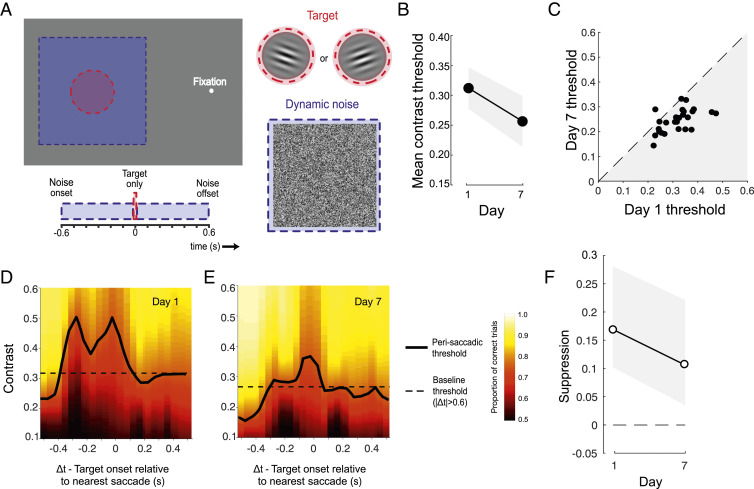
Suppression reduction transfers to untrained spatial locations. (*A*) The Spatial transfer task differed from the main task in two respects: The target was presented to the left (rather than the right) of fixation and subjects indicated whether the grating was ±10° of horizontal (rather than vertical). (*B*) Mean thresholds were lower after training, indicating some transfer of learning. (*C*) All but one subject (represented as dots) had lower thresholds on day 7 than on day 1. (*D* and *E*) Saccadic suppression was still apparent on day 7 but was less than that observed on day 1. (*F*) In the window −100 ms < Δ*t* < 100 ms, suppression was reduced on day 7 relative to day 1. All error bars show 95% CIs.

After verifying that the reduction in suppression was not due to changes in the nature of saccades, we wondered whether the silencing of suppression was specific to the parameters of the trained task, or whether it would generalize to other conditions. A large subset of subjects (*n* = 29) completed two transfer conditions on day 1 and 7. To assess stimulus specificity, target gratings and noise were presented to the left of fixation (as opposed to the right) and targets were rotated by 90° relative to the trained task (Fig. 4*A*). Subjects judged whether the target presented on each trial was rotated clockwise or counterclockwise relative to horizontal while all other characteristics of the stimuli and their timing remained unchanged. In line with previous perceptual learning studies (67–69), we found evidence of partial transfer of learned improvements; on average, contrast thresholds reduced by ∼0.05 (Fig. 4 *B* and *C*), compared with the ∼0.13 reduction achieved by the same subjects in the trained condition (Fig. 1 *B* and *C*). Interestingly, contrast thresholds on trials with perisaccadic target presentations were again elevated on day 1 of testing (Fig. 4*D* and *SI Appendix*, Fig. S4*A*) but were noticeably closer to baseline when evaluated again on day 7 (Fig. 4*E* and *SI Appendix*, Fig. S4*B*). The reduction of saccadic suppression (Fig. 4*F*) was smaller in magnitude than in the trained condition, but remained significant, even after controlling for individual contributions (50.1% attenuation from day 1 to day 7, *P*_2-tailed_ = 0.038). Thus, although suppression was not completely silenced in this condition, it was clearly alleviated by training at a markedly different retinal position and stimulus orientation.

### Silencing of Suppression Is Specific to the Expected Time Window.

**Fig. 5.**
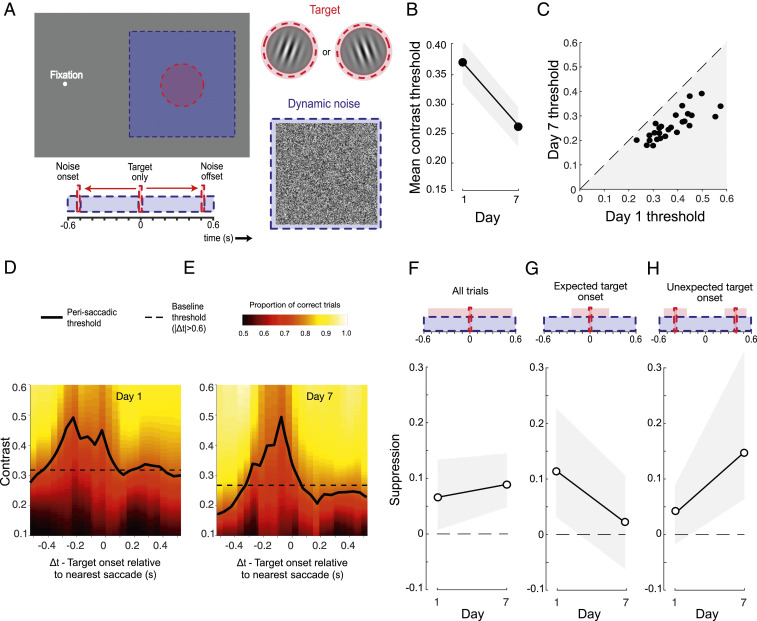
Suppression reduction is tuned to the expected time window. (*A*) The Temporal transfer task differed from the main task in only one respect: The target appeared at a random time within a 1 s interval centred on the expected target onset. (*B*) Mean thresholds were lower after training, indicating some transfer of learning. (*C*) All subjects (represented as dots) had lower thresholds on day 7 than on day 1. (*D* and *E*) Saccadic suppression was of a similar magnitude on day 7 as it was on day 1. (*F*) In the window −100 ms < Δ*t* < 100 ms, suppression marginally increased on day 7 relative to day 1. (*G*) Considering only those trials in which the target occurred near to the expected time, suppression reduction was apparent on day 7 relative to day 1. (*H*) Conversely, suppression increased on day 7 relative to day 1, if only those trials in which the target deviated from the trained onset time are considered. All error bars show 95% CIs.

In a second transfer condition, we tested whether subjects may have learned to temporarily reduce saccadic suppression in time with the expected appearance of the target stimulus. The spatial characteristics of target and noise were retained from the trained task, but target onset time was randomly jittered on each trial within a 1-s interval (Fig. 5*A*). In this condition, we again saw partial transfer of learning, with some improvement in thresholds from day 1 to day 7 (Fig. 5*B*) observed in all subjects (Fig. 5*C*). Robust elevations of perisaccadic thresholds were also evident on day 1 (Fig. 5*D* and *SI Appendix*, Fig. S5*A*), consistent with other conditions. However, in this instance, there was no systematic change in the perisaccadic threshold profile by day 7 (Fig. 5*E* and *SI Appendix*, Fig. S5*B*) and no reduction in saccadic suppression (Fig. 5*F*, *P*_2-tailed_ = 0.46). The importance of target timing in this condition is reinforced if we separately analyze trials according to when the target appeared. For those trials when the target occurred within 240 ms of the expected (i.e., trained time), there is some evidence of a reduction in suppression on day 7, although with noisier threshold estimates the change does not reach statistical significance (Fig. 5*G*, 81.4% attenuation from day 1 to day 7, *P*_2-tailed_ = 0.053). In contrast, when the target appeared more than 240 ms from the expected time, suppression was significantly greater on day 7 than it had been on day 1 (Fig. 5*H*, *P*_2-tailed_ = 0.022). This implies that changes in saccadic suppression are tuned around the expected onset of the target stimulus.

## Discussion

Repeated training on a task requiring detection of brief visual stimuli led to a striking change in the magnitude of saccadic suppression. Within 5 days of training (around 2,500 trials on the main task), suppression dropped from an initial ∼50% elevation of contrast thresholds to being statistically indistinguishable from zero. Careful balancing of the contributions from each subject revealed that this silencing of suppression was not due to individual differences in sensitivity and saccade frequency. Nor was it a by-product of training-related changes in the rate of saccades or their characteristics.

Previous work has demonstrated that perceptual learning is possible both before (48) and during (49) saccades. Porat and Zohary (49) demonstrated that shape discrimination could improve, even if the stimulus was presented during a saccade. In the same study, perisaccadic spatial mislocalizations of the stimulus were robust to learning, leading the authors to conclude that the perceptual consequences of saccades are resistant to top-down modulations. However, recent work has challenged this notion, revealing that suppression of the displacement of a stimulus during a saccade can change based on stimulus context (70). Our data provide a direct demonstration that saccadic suppression of sensitivity can be modified and that this decrease in suppression generalizes to untrained locations and tasks. The reduction was neither specific to the spatial location of the stimulus nor the nature of the task (horizontal vs. vertical judgments), although transfer of the effect to new spatial locations was not complete. Similarly, behavioral learning transferred to the novel spatial location, but not completely, which is in accordance with previous work using a similar task (58). Spatial transfer of suppression reduction suggests a mechanism that acts generally across eye movements rather than being specific to the trained retinal location.

Suppression reduction operated over a restricted temporal window tied to the time at which the target was expected to appear. This strong dependence on timing is reassuring, as shutting down suppression completely would likely have serious consequences for visual stability. Instead, our results suggest that suppression can be temporarily silenced when behavioral demands dictate that it is advantageous to do so. In the task employed here, the timing of both the noise and the target were constant relative to a subject’s response, which led to a rhythmic, temporally proximal sequence, a recipe that is beneficial for strong learning (71). Saccade production was also tightly coupled to event timing, with a marked reduction in saccades around target onset (refer to Fig. 3*A*), which indicates anticipation of target timing (66, 72, 73). Anticipation is a strong driver of the allocation of temporal attention (for a review, see ref. 74), providing a mechanism that putatively links the oculomotor behavior, perceptual learning, and reduction of saccadic suppression reported here.

The relative contribution of active and passive mechanisms to saccadic suppression is unclear. While it is likely that passive mechanisms dominate under lighted, contoured viewing conditions, suppression is still present even after controlling for masking effects, implying that an active mechanism also exists (9, 75). Masking can be minimized using a blank background; however, we chose to use a noise task to maximize learning (58), and so did not control for masking effects. Could suppression reduction be due to a decrease in the effectiveness of backward masking by the structured postsaccadic image? There is evidence that learning occurs for a variety of backward masking tasks, including object identification (76, 77), letter identification (78), and metacontrast masking (79, 80); however, we are unaware of any study that has demonstrated learning in a contrast detection task, like the one used here, solely with backward masking. A significant contribution to learning in these tasks comes from filtering of the masking noise, as it becomes more familiar with training: Changing the nature of the noise leads to an increase in thresholds back toward prelearning levels (78). Neurophysiological evidence from monkeys suggests that this noise filtering could be due to a rebalancing of neural activity, with a reduction in inferior temporal cortical responses to the mask leading to enhanced processing of the target (81). A similar mechanism could be responsible for the reduction in saccadic suppression reported here, with training leading to a dampening or reweighting of saccade-related masking.

Suppression reduction could also be due to an adaptation of active suppression mechanisms. The superior colliculus (SC) has an important role in saccade generation (82, 83) and is also thought to be the source of the corollary discharge (84). The pathway from SC to the frontal eye fields (FEFs) via the medial dorsal nucleus of the thalamus (85), is the prime candidate for the projection of the corollary discharge for use by sensory cortex (86). A reduction in saccadic suppression has two possible causes. Either the corollary discharge signal itself is modulated as the result of learning or the readout of this signal is adapted at some stage of processing. The corollary discharge is posited to be a faithful description of the eye movement and should thus only change with features of the saccade, such as amplitude. For example, in saccadic adaptation experiments, the perceived position of the shifted target dot is relative to the saccade, either unadapted or adapted (35, 87), providing evidence that the corollary discharge is a record of the statistics of the eye movement (75). We noted only a slight decrease in saccade amplitude across the week, implying that the corollary discharge would not have changed markedly and suggesting that the silencing of suppression is due to an adaptation of the readout of the corollary discharge. As discussed above, the FEF and medial dorsal nucleus of the thalamus are potential locations for the modulation of the corollary discharge. However, taking into account the strong temporal dependency discussed previously, the striatal dopaminergic system or the left intraparietal sulcus, which are implicated with temporal expectation (88, 89) and temporal attention (90, 91), respectively, are more suitable candidates.

Studies into saccadic suppression have generally investigated suppression during large, voluntary saccades rather than spontaneous saccades made during fixation. The overwhelming evidence is that fixational saccades, rather than being idiosyncratic eye movements specialized for fixation, are on the lower end of a continuum that includes large voluntary saccades, underlying a common visual sampling strategy. The majority of research has emphasized the commonalities between fixational saccades and large voluntary saccades. Both types of eye movement are generated in the SC from the same spatial map (55), occur while brainstem omnipause neuron responses are paused (92, 93), are generally binocular and conjugate (94, 95), have similar spatiotemporal characteristics across viewing tasks (43), have a main sequence relationship between amplitude and velocity (96), and function to position gaze on to specific regions of the retina at different spatial scales (97). Furthermore, suppression during fixational saccades is broadly similar to that during large voluntary saccades, with a similar dependence on the spatial content of the stimulus (12, 25), a similar duration (26, 54), and similar modulations of visual cortex responses (98), albeit with a magnitude scaled to the size of the eye movement, at least for centrally presented stimuli (12). In addition, the perisaccadic compression of space and time that has been reported for large voluntary saccades (52, 99) has also been demonstrated for fixational saccades (50, 53). The body of evidence thus suggests that the visual consequences of fixational and voluntary saccades are comparable.

A common challenge faced by our sensory systems is to disambiguate self-generated, reafferent sensations from externally generated, exafferent sensations. A ubiquitous solution to this problem is to suppress reafferent signals (100–102). Maintenance of visual stability is one example where this approach is applied: Suppression occurs not only around saccades, but also blinks (103, 104), dynamic changes in ocular accommodation (105), and vergence eye movements (106). These types of visual suppression share features, such as their time course, magnitude, and spectral dependency, suggesting common underlying mechanisms (107). Examples of reafferent suppression in other modalities include the inability to tickle oneself, and the dampening of self-generated sounds during movement (108). By suppressing neural responses to predictable self-movement–generated sounds that would act as a strong stimulus for the ear, such as speech (101), the auditory system becomes more sensitive to other environmental signals. While suppressing reafferent signals is functionally beneficial most of the time, there are situations where it is advantageous to release this suppression. One instance is during learning to speak (or to sing), where close monitoring of the unadulterated reafferent signal is advantageous. Interestingly, while voicing generally suppresses cortical activity in primates, the reafferent effects of singing in songbirds, one of the few nonhuman species that demonstrate vocal learning, are predominantly excitatory (108), suggesting that suppression is actively reduced. How humans learn to speak or to sing is unclear, but a system in which reafferent suppression can be flexibly modulated during acquisition of learned behavior offers clear advantages. Our data reveal that such a system exists for saccade-related visual suppression, providing a flexible strategy that could be applied more generally across the senses.

## Materials and Methods

### Subjects and Ethics Approval.

Forty-four individuals (23 females: mean age, 25; range, 16 to 50) participated in the main experiment, and from these individuals, a subset of 29 (15 females: mean age, 25; range, 16 to 50) also completed the transfer conditions. All were naive to the purpose of the experiment, with the exception of two authors (N.W.R. and P.V.M.) who sat as subjects. All subjects had normal or corrected-to-normal vision. The study was approved by the School of Psychology Ethics Committee at the University of Nottingham. Subjects provided written consent and were reimbursed for their participation.

### Stimulus Materials and Procedure.

#### Main experiment.

Subjects sat in a dark room and were instructed to maintain fixation on a central white dot (2 pixels = 0.06° diameter; Weber contrast, 0.99). The head was secured using a chin and forehead rest. Target stimuli were Gabor patches (carrier spatial frequency = 2 c/deg; envelope SD = 5/6°; 1 frame duration) presented 6° to the right of fixation. Carrier phase was randomized to prevent the buildup of a retinal afterimage and orientation was randomly set to ±10° of vertical. Pilot testing confirmed that, with these orientations, task performance was limited by contrast detection rather than orientation discrimination. Target stimuli were presented in between dynamic Gaussian noise fields (10 × 10°; SD = 0.33; 50 frames in duration and updated on each frame) centered at the same eccentricity as the Gabor.

Stimuli were generated using PsychoPy (109, 110) and displayed on a 20-inch CRT monitor (Iiyama Vision Master Pro-514; resolution, 1,024 × 768; refresh rate, 85 Hz; viewing distance, 75 cm; background luminance, 45 cd/m^2^). The luminance response of the monitor was gamma-corrected and 14-bit grayscale resolution was obtained using a Bits++ stimulus processor (CRS, Ltd.). Stimulus contrast was adaptively varied using a one-up three-down staircase with a starting Michelson contrast of 0.89 and fixed step size of 0.1 log units. Subjects indicated the orientation of the Gabor (±10° of vertical) using the left and right arrow keys. The next trial started immediately after the subject had responded. Staircases continued until at least eight reversals and 100 trials had occurred. To ensure maintained fixation, eye movements were monitored online and any trials in which eye position deviated from fixation by at least 3° were repeated. Any blinks (and the eye data 100 ms before and after) were disregarded during this monitoring process. A high-pitched tone (1,000 Hz, 100 ms) was presented after a correct response, a low-pitched tone (500 Hz, 100 ms) was presented after an incorrect response, and a white noise burst (300 ms) was presented after a trial, which was to be repeated. On each of 7 consecutive days (not including weekends), subjects performed five repeats of the staircase procedure, resulting in 500 trials per day (not including repeated trials, which were discarded from any subsequent analyses).

#### Transfer experiments.

The transfer experiments were identical to the main experiment during the training phase (days 2 to 6) but had additional tasks on days 1 and 7. There were two transfer conditions (Spatial and Temporal), which were the same as the main experiment apart from the following differences. In the Spatial task, the Gabor and noise were presented 6° to the left, rather than right, of fixation, and the task was to indicate whether the Gabor was oriented ±10° of horizontal, rather than vertical, using the up/down arrow keys. In the Temporal task, the timing of the target was randomized on each trial so that it could appear at any time within a 1-s window around the trained target time (specifically, the target could randomly appear at any time from 82 ms after noise onset to 82 ms before noise offset). Subjects completed five staircase repetitions (one block) of each transfer condition, along with the main task block, on days 1 and 7. The order in which the three task blocks were completed was counterbalanced across subjects, with the same order used by each subject on days 1 and 7.

#### Eye movement analysis.

Eye movements were recorded binocularly (500 Hz) with an Eyelink 1000 infrared eye tracker (SR Research). Raw gaze positions were converted to degrees of visual angle using the data from a nine-point calibration at the beginning of each staircase. To reduce blink artifacts, subjects were encouraged to restrict blinking to the period between a stimulus occurring and their response. Data during blink periods (pupil size = 0), along with a buffer of samples 200 ms before and after, were removed for saccade detection (see below). Two separate analyses confirmed that our results were not due to changes in blinking behavior across the week. Suppression reduction remained even when all trials in which the subject blinked within the suppression window (defined below: −100 to 100 ms relative to stimulus onset) or within the noise window (−600 to 600 ms relative to stimulus onset) were removed (*SI Appendix*, Fig. S6).

Saccades were detected using an established velocity-threshold algorithm (62, 111), using a threshold of six times the SD of the median velocity. Identified saccades with duration <6 ms or amplitude <3 arcmin were discarded. Saccades within 50 ms of each other were merged to deal with situations in which overshoots were classified as separate saccades. Saccades were required to overlap in time across both eyes, which improved the robustness of saccade classification. We verified that fixational saccades followed the main sequence (29) by plotting amplitude against peak velocity. For all saccades across the population, *R* was equal to 0.94, ranging from 0.89 to 0.99 across individuals. In total, 211,351 trials were analyzed.

#### Threshold computation and statistical tests.

Psychophysical data were initially screened to identify and remove sessions containing nonconverging staircases. Specifically, we removed any session in which half or more of all trials were confined to the two highest contrast levels in the staircase (0.71 and 0.89). On the main task, this accounted for 4% of sessions, the majority of which were on the first 2 days in a small number of subjects (*SI Appendix*, Fig. S7). This step was important to increase the robustness of psychometric function fitting but may have resulted in a slight underestimation of pretraining thresholds (and, consequently, overall learning effects).

To quantify learned improvements in performance, we took the standard approach of computing individual thresholds for each subject using all trials of a given condition completed on each day. Note that, due to data screening, this was not possible on day 1 for a small number of subjects (three on main task, three on Spatial transfer task, one on Temporal transfer task), so learning effects were calculated from the remaining subjects in these conditions (*n* = 41 in Figs. 1 *B* and *C* and 4 *B* and *C*; *n* = 43 in Fig. 5 *B* and *C*). Trials were used to form a psychometric function relating the proportion of correct responses to stimulus contrast, which was fitted using a maximum likelihood criterion with a cumulative Gaussian function of the following form:

p(c)=0.5+(1−λ)∗0.5∗normcdf(c,x,σ),

where λ is the lapse rate, x is the contrast at which the point of inflection occurs, and σ controls the slope of the function. Threshold was defined as the contrast corresponding to 75% correct performance. Mean thresholds were calculated for each day, with 95% CIs calculated from between-subject variability.

To assess perisaccadic changes in performance, we first calculated the time difference between stimulus onset and the onset of the nearest microsaccade on each trial (Δ*t*). This metric was then used to group trials from all subjects into 0.15-s-wide time bins centered between Δ*t* = −0.525 s and Δ*t* = 0.525 s, along with a baseline bin, in which no saccade occurred within 0.6 s of the target (|Δ*t*| > 0.6). Thresholds were calculated for each bin in the manner described above. Statistical comparison of perisaccadic thresholds to baseline is described and reported in *SI Appendix*, Figs. S2, S4, and S5. To derive a single measure of saccadic suppression on each day, thresholds were computed using a broad perisaccadic window (−0.1 s <Δ*t* < 0.1 s) and baseline corrected. The 95% CIs were calculated using nonparametric bootstrapping, resampling across trials prior to binning (1,000 samples). To assess the statistical significance of changes in suppression across the week, nonparametric permutation tests were conducted by resampling trial data after random shuffling of day 1 and day 7 labels. Importantly, this was done in a manner that preserved the number of trials contributed by individual subjects to each measure. In each case, two-tailed *P* values were calculated, reflecting the proportion of permuted absolute suppression changes (|Suppression_day__1_ − Suppression_day__7_|) that exceeded the observed absolute suppression change. Percentage attenuation values were calculated according to the following formula: 100*(Suppression_day__1_ − Suppression_day__7_)/Suppression_day__1_.

A resampling approach was also used to assess whether changes in saccade statistics might have affected suppression across the week. For example, in order to assess the effect of saccade rate, the numbers of saccades falling within suppression (−0.1 s < Δ*t* < 0.1 s) and baseline (|Δ*t*| < 0.6 s) windows on each day were first calculated. Then, for each day, those numbers of trials were randomly drawn from data pooled across the entire week, and the difference between baseline and suppression thresholds was computed. This is equivalent to shuffling day labels and computing the distribution of threshold differences while maintaining the number of suppression and baseline trials on each day. Likewise, the contribution of saccade orientation or amplitude changes to suppression was assessed by resampling while matching the number of trials with leftward/rightward saccades or the number of trials with saccades within five equally populated amplitude ranges for each day. Predicted suppression profiles in Fig. 3 *D*, *H*, and *L* show the distribution (mean and 95% confidence limits) across 1,000 resampled analyses).

All analyses were done in MATLAB (MathWorks) using custom-written software.

## Supplementary Material

Supplementary File

## Data Availability

Trial-by-trial log of stimulus parameters, psychophysical responses, and saccade characteristics have been deposited in Open Science Framework (https://osf.io/xzphc/?view_only=0651322c02ff450ba22a8d0011aea480).
