# Supplementary material for: Learning to silence saccadic suppression
Source: Proc Natl Acad Sci U S A. 2021 Feb 1;118(6):e2012937118. doi: 10.1073/pnas.2012937118 (PMC8018005; doi:10.1073/pnas.2012937118)
Supplement: Supplementary File [file pnas.2012937118.sapp.pdf]

PNAS
www.pnas.org

Supplementary Information for:

Learning to silence saccadic suppression

Chris Scholes*, Paul V. McGraw & Neil W. Roach*

* Correspondence to Neil W. Roach or Chris Scholes
Email: neil.roach@nottingham.ac.uk or chris.scholes@nottingham.ac.uk

This PDF file includes:

- Figures S1 to S8
- Supplementary text
- SI References

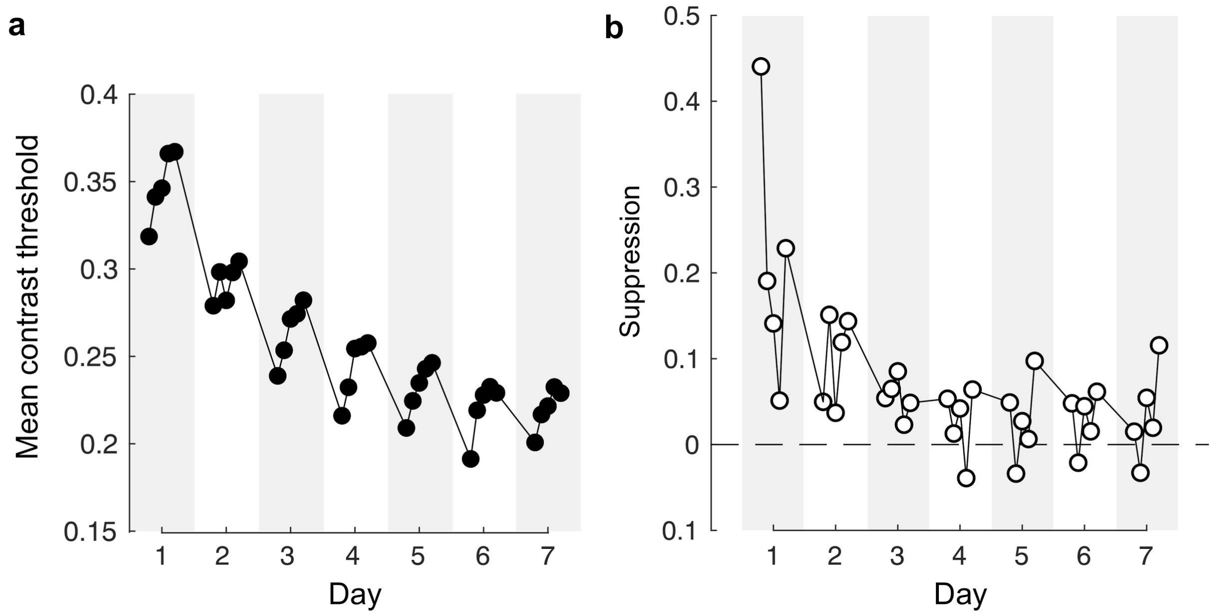

Figure S1. Within- and between-day dynamics of perceptual learning and attenuation of suppression. **a** Behavioural thresholds exhibited within-day increases and between-day decreases, characteristic of perceptual learning. **b** Although there was a clear reduction in the magnitude of suppression across days, within-day changes were less systematic than for behavioural thresholds.

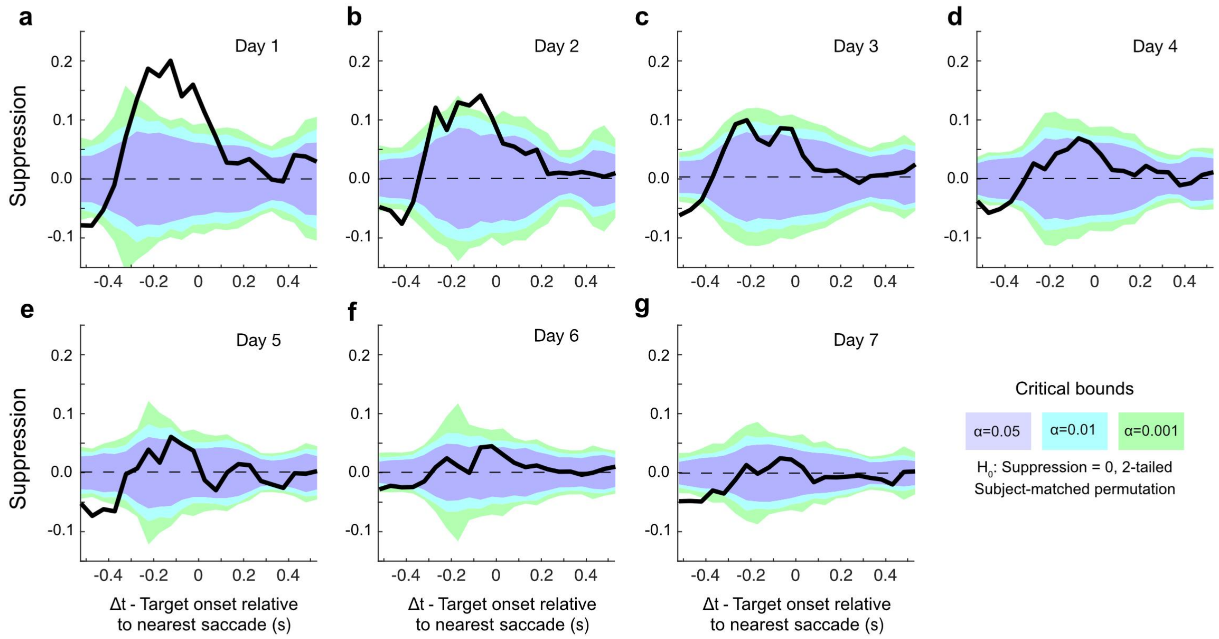

Figure S2. a-g Assessing the statistical significance of saccadic suppression profiles on the trained task. Each plot shows the suppression profile for a given day, calculated by subtracting baseline thresholds from the perisaccadic thresholds shown in Figure 2a-g. Colored contours show critical bounds for 2-tailed permutation tests assessing the null hypothesis that suppression is zero for a range of alpha-levels. Importantly, permutations were done in a manner that preserved the number of trials contributed by each subject to threshold estimates. Specifically, we first calculated the number of trials that each subject contributed to a given time bin and the baseline. Corresponding numbers of trials were then randomly drawn (with replacement) from all trials for that subject for that day, and the difference between the baseline threshold and the threshold for that time bin was computed. This is equivalent to shuffling the Δt labels of all trials and addressing how likely it is to achieve the observed threshold difference for that time bin, with the observed contribution of trials from each individual.

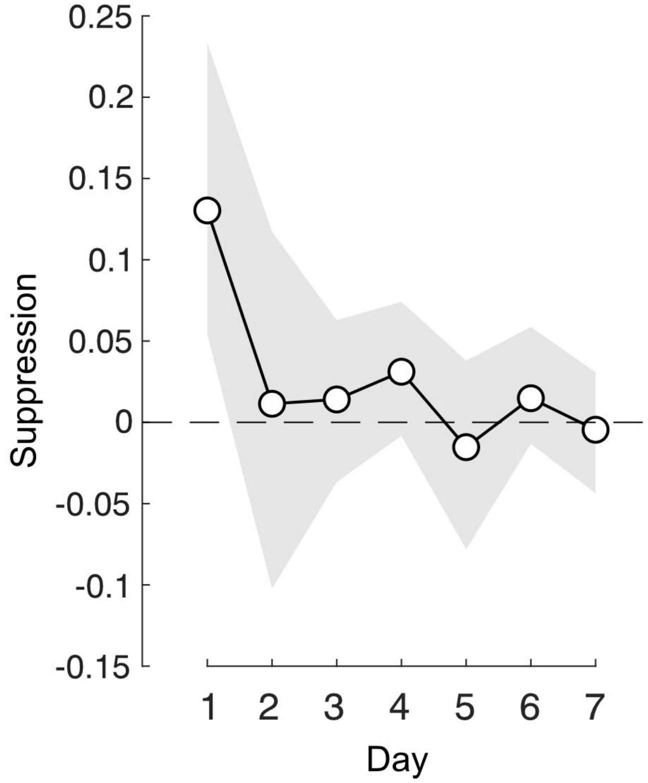

Figure S3. Suppression reduction was observed when only trials with pre-stimulus saccades (0ms < Δt < 100ms) were considered, confirming that the effect was not a by-product of changes in the stimulus-related rate-signature.

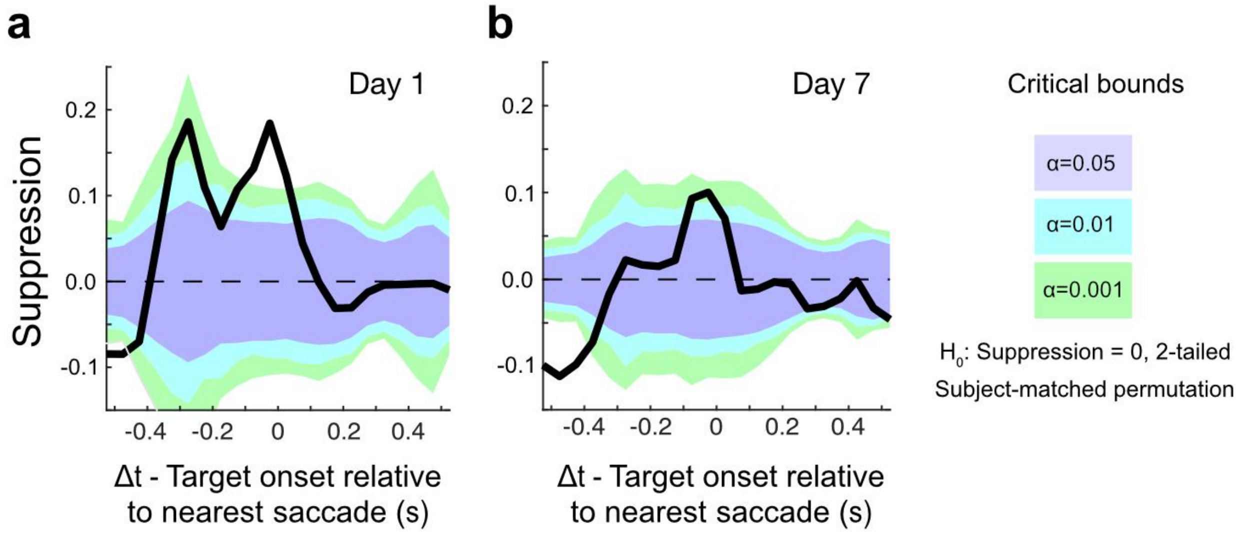

Figure S4. a-b Assessing the statistical significance of saccadic suppression profiles obtained at an untrained spatial location and orientation. Each plot shows the suppression profile for a given day, calculated by subtracting baseline thresholds from the perisaccadic thresholds shown in Figure 4d-e. Colored contours show critical bounds for 2-tailed permutation tests assessing the null hypothesis that suppression is zero for a range of alpha-levels. Importantly, permutations were done in a manner that preserved the number of trials contributed by each subject to threshold estimates. Specifically, we first calculated the number of trials that each subject contributed to a given time bin and the baseline. Corresponding numbers of trials were then randomly drawn (with replacement) from all trials for that subject for that day, and the difference between the baseline threshold and the threshold for that time bin was computed. This is equivalent to shuffling the Δt labels of all trials and addressing how likely it is to achieve the observed threshold difference for that time bin, with the observed contribution of trials from each individual.

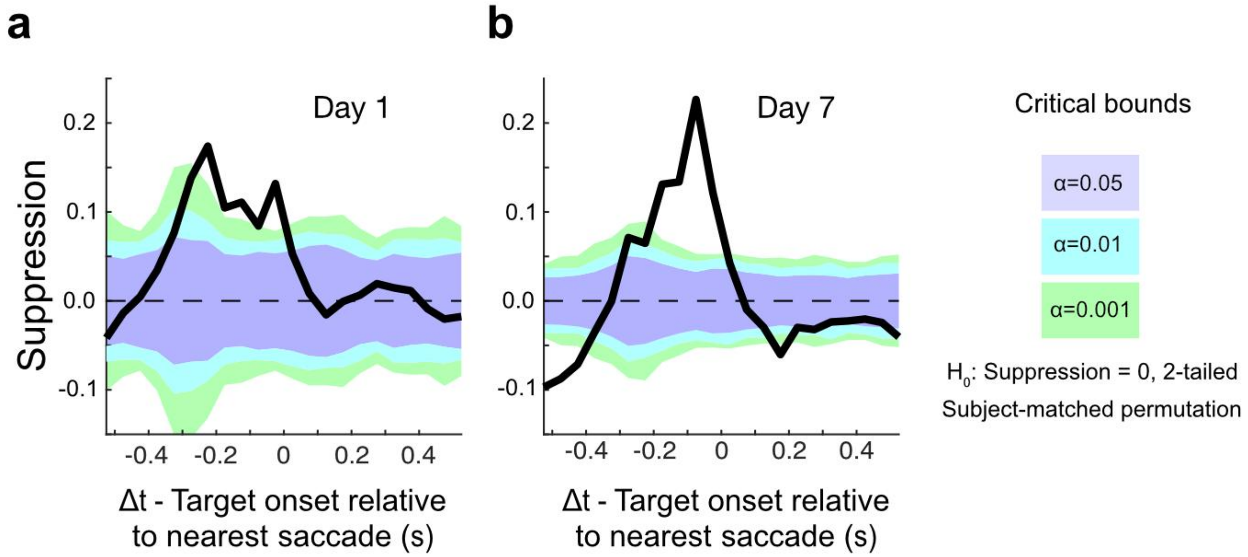

Figure S5. a-b Assessing the statistical significance of saccadic suppression profiles obtained with randomly jittered target onset. Each plot shows the suppression profile for a given day, calculated by subtracting baseline thresholds from the perisaccadic thresholds shown in Figure 5d-e. Colored contours show critical bounds for 2-tailed permutation tests assessing the null hypothesis that suppression is zero for a range of alpha-levels. Importantly, permutations were done in a manner that preserved the number of trials contributed by each subject to threshold estimates. Specifically, we first calculated the number of trials that each subject contributed to a given time bin and the baseline. Corresponding numbers of trials were then randomly drawn (with replacement) from all trials for that subject for that day, and the difference between the baseline threshold and the threshold for that time bin was computed. This is equivalent to shuffling the Δt labels of all trials and addressing how likely it is to achieve the observed threshold difference for that time bin, with the observed contribution of trials from each individual.

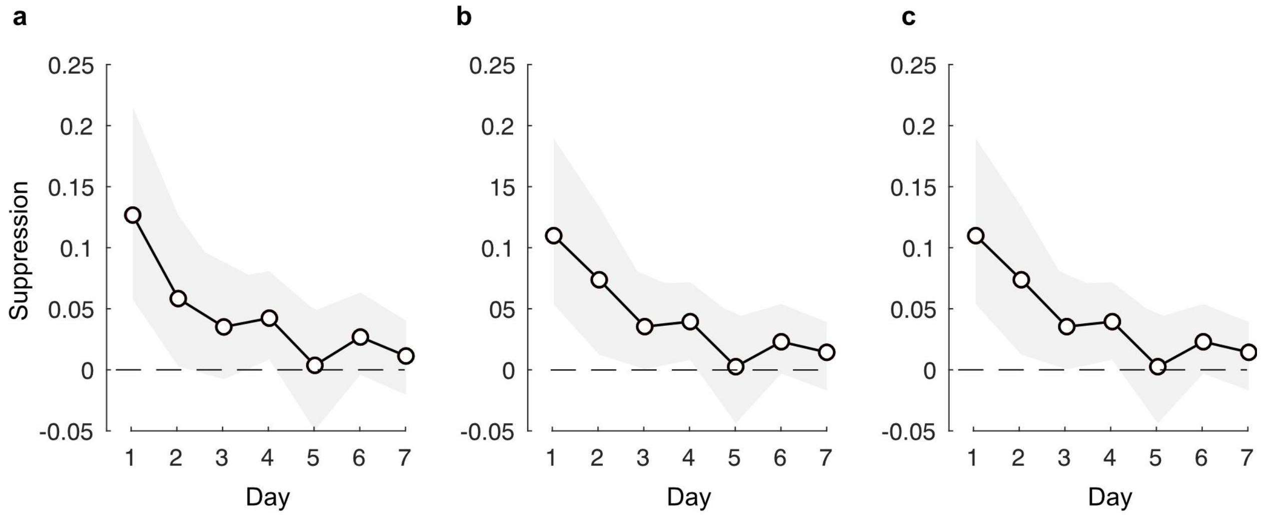

Figure S6. Suppression reduction was similar after removal of trials with blinks. **a** Suppression magnitude, computed from the broad suppression window (-100ms < Δt < 100ms) minus the baseline threshold, including all trials (duplicated from Figure 2h). **b** Suppression magnitude when trials with a blink during the suppression window were removed from the analysis. **c** Suppression magnitude when trials with a blink during the noise window (-600ms < Δt < 600ms) were removed from the analysis.

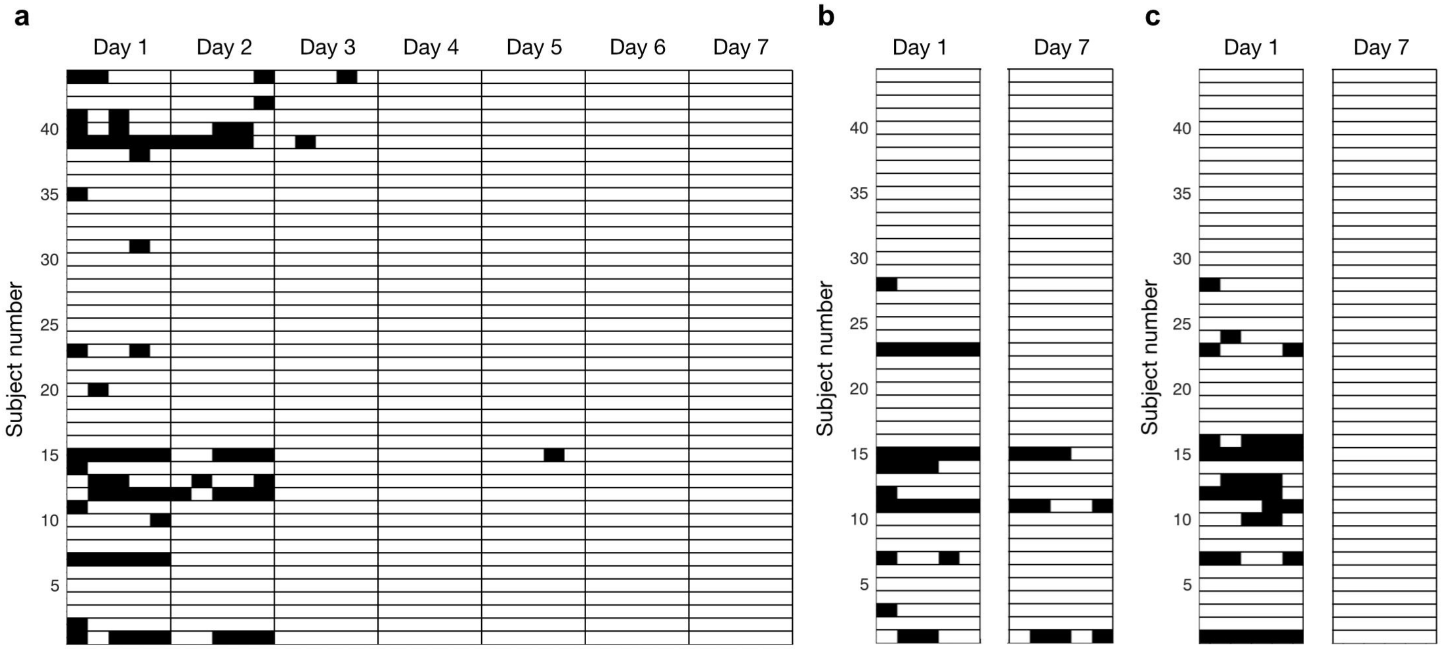

Figure S7. Sessions in which staircases did not converge were removed from subsequent analysis. Removed sessions for each subject are indicated by black markers for each Day. **a** The main training task. **b** The *Spatial* transfer task. **c** The *Temporal* transfer task.

Supplementary Text 1.

While the focus of our study was suppression of contrast sensitivity within 100ms of saccade onset ($-0.1s<\Delta t<0.1s$), two additional features of the pre-saccadic threshold changes warrant mentioning: (i) impairments in sensitivity often appeared to extend up to ~300ms prior to saccade onset; (ii) sensitivity appeared to be *enhanced* when target stimuli preceded movement of the eye by 400-600ms. Here we describe and consider potential explanations for these features.

(i) Performance-linked modulation of the saccadic rate signature

The timing of these effects raises uncertainty about the causal relationship between saccades and task performance. While it is possible that they reflect genuine changes in sensitivity, it is also possible that they are caused by performance-linked change in the probability of subjects making a saccade after target onset. Specifically, if subjects are less likely to make a saccade 200-300ms after target onset when they have successfully detected it, the selective analysis of trials with this saccade timing would likely yield less correct responses and a higher threshold estimate. Likewise, an increase in the probability of making saccades 400-600ms after target onset following successful detection, would yield a lower threshold estimate. This is plausible, given that brief visual presentations are known to trigger a modulation in saccade rate during attempted fixation (i.e. the rate signature) that is dependent on visibility[5,6]. Therefore, caution is required when interpreting any performance changes linked exclusively to spontaneous saccades occurring *after* stimulus onset. To avoid any uncertainty over the interpretation of our results, we therefore restricted our analyses to a narrow peri-saccadic window ($-0.1s<\Delta t<0.1s$) and were careful to ensure that our results remained robust even if the pre-saccadic interval was excluded entirely ($0<\Delta t<0.1s$, Figure S3).

(ii) Oscillations of visual sensitivity

Recent studies suggest that saccadic suppression may be embedded within a broad periodic oscillation of visual sensitivity over time[1–4]. For example, Benedetto & Morrone[2] measured subjects' ability to detect contrast increments presented as they made sequential saccades between two locations. They found oscillations in performance time-locked to saccadic onset, starting at least a second prior to the saccade. While our sensitivity increase falls within this temporal interval, features of our dataset are less consistent with this explanation. Oscillations in performance are typically reported to extend for some time after the saccade. However, we find no systematic sensitivity enhancement following the period of saccadic suppression. Moreover, oscillations in performance have been reported in the delta-band (2-3Hz) for large saccades[2,3], extending into the alpha- and beta-bands (8-20Hz) for fixational saccades[4]. In contrast, we found only one asymmetric period of suppression and enhancement within a 1.2s epoch, making it unlikely we are measuring a similar mechanism to these earlier studies.

(iii) Release of visual masking

A final possible explanation for the apparent increase in sensitivity relates to the fact that visual target stimuli were embedded within a larger period of contrast noise. Contrast masks are an effective means of impairing visual performance, particularly when presented after the target stimulus (i.e. backwards masking). It is possible that suppression induced by saccades after target onset might reduce the effective contrast of the backwards mask, rendering it less effective. Consistent with this explanation, the epoch in which an increase in sensitivity was found corresponds to saccades occurring 400-600ms after target onset (during the final 200ms of the post-stimulus mask). This specific timing could reflect the requirement for saccades to be sufficiently delayed relative to target onset to avoid compromising the visibility of the target itself.

Supplementary Text 2.

Here we provide a detailed description of a permutation test used to assess the reduction in saccadic suppression from Day 1 to Day 7 that explicitly controls for the redistribution of saccade parameters. Table S1 shows the number of saccades falling within suppression (-0.1s<Δt<0.1s) and baseline (|Δt|<0.6s) windows, split by direction and amplitude on each day. On each iteration of the permutation test, we randomly shuffled the 'Day1' and 'Day7' labels of trials falling within each direction/amplitude combination (i.e within each shaded table cell), then re-calculated the magnitude of the change in suppression between Day 1 and Day 7. Note, this approach resamples the data under the null hypothesis that training does not affect suppression (i.e. Day 1=Day 7), whilst maintaining the joint saccade statistics on each day.

**Suppression window** -0.1s<Δt<0.1s	Amplitude **<15arcmin**	Amplitude **15-23arcmin**	Amplitude **23-34arcmin**	Amplitude **34-52**	Amplitude **>52arcmin**	
Directed ***away*** from target ± 45deg	Day1/Day7 37/40	Day1/Day7 75/53	Day1/Day7 87/8533	Day1/Day7 132/95	Day1/Day7 198/99	Day1/Day7 529/340
Directed ***towards*** target ± 45deg.	Day1/Day7 44/57	Day1/Day7 49/75	Day1/Day7 96/96	Day1/Day7 144/99	Day1/Day7 324/251	Day1/Day7 657/578
	Day1/Day7 81/97	Day1/Day7 124/128	Day1/Day7 183/149	Day1/Day7 276/194	Day1/Day7 522/350	Day1/Day7 1186/918

**Baseline window** |Δt|<0.6s	Amplitude **<15arcmin**	Amplitude **15-23arcmin**	Amplitude **23-34arcmin**	Amplitude **34-52**	Amplitude **>52arcmin**	
Directed ***away*** from target ± 45deg	Day1/Day7 462/511	Day1/Day7 636/893	Day1/Day7 907/1112	Day1/Day7 1125/1294	Day1/Day7 1338/1362	Day1/Day7 4468/5172
Directed ***towards*** target ± 45deg.	Day1/Day7 493/557	Day1/Day7 475/683	Day1/Day7 488/657	Day1/Day7 627/737	Day1/Day7 888/816	Day1/Day7 2971/3450
	Day1/Day7 955/1068	Day1/Day7 1111/1576	Day1/Day7 1395/1769	Day1/Day7 1752/2031	Day1/Day7 2226/2178	Day1/Day7 7439/8622

Table S1. Number of trials containing a saccade within the Suppression window (-0.1s<Δt<0.1s) and Baseline window (|Δt|>0.6s), broken down by saccade direction and amplitude. Separate counts are shown for Day 1 and Day 7 within each cell.

Figure S8 shows the resulting sampling distribution for the change in suppression from Day 1 to Day 7, based on 10,000 permutations, along with the value observed in our original analysis. This yielded a 2-tailed p-value of 0.0184. We can therefore be confident that the change in suppression is not a secondary consequence of changes in saccade parameters.

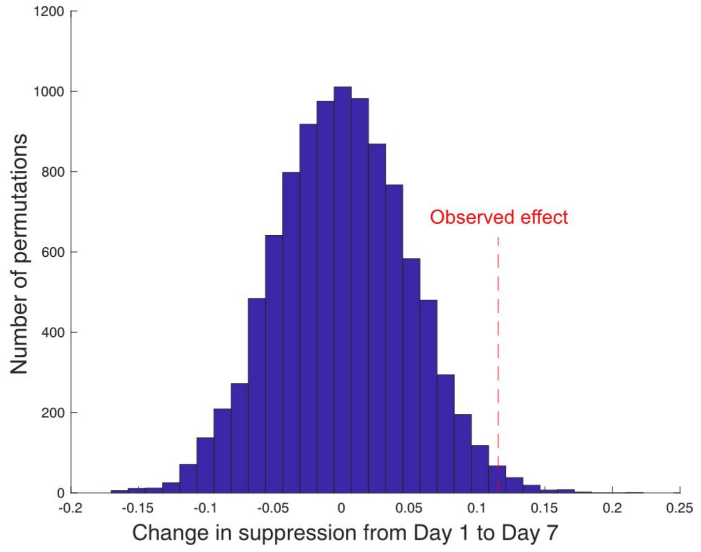

Figure S8. Sampling distribution of permutation test testing null hypothesis that suppression is equivalent on Day 1 and Day 7, but controlling for changes in the number, direction and amplitude of saccades on each day.

SI References

1. Benedetto, A., Morrone, M. C. & Tomassini, A. The Common Rhythm of Action and Perception. *J. Cogn. Neurosci.* 1–14 (2019) doi:10.1162/jocn_a_01436.
2. Benedetto, A. & Morrone, M. C. Saccadic Suppression Is Embedded Within Extended Oscillatory Modulation of Sensitivity. *J. Neurosci. Off. J. Soc. Neurosci.* **37**, 3661–3670 (2017).
3. Hogendoorn, H. Voluntary Saccadic Eye Movements Ride the Attentional Rhythm. *J. Cogn. Neurosci.* **28**, 1–11 (2016).
4. Bellet, J., Chen, C.-Y. & Hafed, Z. M. Sequential hemifield gating of α- and β-behavioral performance oscillations after microsaccades. *J. Neurophysiol.* **118**, 2789–2805 (2017).
5. Scholes, C., McGraw, P. V., Nyström, M. & Roach, N. W. Fixational eye movements predict visual sensitivity. *Proc. R. Soc. B Biol. Sci.* **282**, 20151568 (2015).
6. White, A. L. & Rolfs, M. Oculomotor inhibition covaries with conscious detection. *J. Neurophysiol.* **116**, 1507–1521 (2016).
